# Possible relation between consumption of different food groups and depression

**DOI:** 10.1186/s40359-019-0292-1

**Published:** 2019-03-06

**Authors:** G. Grases, M. A. Colom, P. Sanchis, F. Grases

**Affiliations:** 1 Centro de Enseñanza Superior Alberta Jiménez (CESAG), 07013 Palma de Mallorca, Spain; 2 Psycology and Neurology Center (CLONUS), 07014 Palma de Mallorca, Spain; 3 University Institute of Health Sciences Research (IUNICS- IdisBa), University of Balearic Islands, Carretera Valldemossa Km 7,5, 07122 Palma de Mallorca, Spain

**Keywords:** Food, Depression, Precursors to neurotransmitters, Oxidative stress

## Abstract

**Background:**

Diverse studies have investigated the relationship between diet and depression. In fact some cross-sectional studies suggested that a healthy diet reduced the risk for depression. The main objective of this study was to assess the relationship of consumption of different food groups with depression. The food groups were selected based on their content of substances that were precursors to neurotransmitters (tryptophan or inositol) or their effect on oxidative stress.

**Methods:**

This observational retrospective study compared the diets of individuals who were with depressive symptoms (Beck Depression Inventory Questionnaire [BDI] ≥ 10; 53 women, 23 men, age 38+/− 11) and with no depressive levels (BDI < 10; 33 women, 23 men, age 41+/− 13). Dietary data were collected from a questionnaire that asked about consumption of legumes, nuts, whole-grain foods, fruits and vegetables, chocolate, and sweet foods and refined sugars.

**Results:**

Depressed individuals consumed significantly lower amounts of legumes, fruits, and vegetables, but higher amounts of sweets and refined sugars (*p* < 0.05 for all comparisons). After statistical adjustment for age and sex, the consumption of no legumes (adjusted odds ratio [aOR] = 2.60, 95% confidence interval [CI] = 1.19–5.67), low consumption of fruits and vegetables (aOR = 2.69, 95% CI = 1.18–6.13), and high consumption of sweet foods and refined sugars (aOR = 1.91, 95% CI = 1.23–2.99) were significantly associated with depression. The two groups had no significant differences in the consumption of chocolate.

**Discussion:**

The results indicate significant relationships of the consumption of certain foods with depression, although the study design precludes any conclusions regarding causality. Further studies are necessary to determine the causal relationships of the consumption of specific foods with depression, and of depression with the consumption of specific foods.

**Conclusion:**

In spite of the limitations, we find that individuals without depression consumed more legumes, fruits, and vegetables, but fewer sweets and pastries than those with depression.

**Electronic supplementary material:**

The online version of this article (10.1186/s40359-019-0292-1) contains supplementary material, which is available to authorized users.

## Background

The data on prevalence of depression in Spain varied from 1.12% in preschool children, 8.56% in the general population and up to 55.6% in university students [1]. There are significant positive associations of depression with physical and other mental illnesses, the use of recreational drugs, and suicidal behaviors. Thus, depression is a major public health problem, and multi-disciplinary study of depression is necessary to develop methods that reduce the symptoms and prevent its devastating effects.

Several studies have investigated the relationship between diet and depression. In particular, some cross-sectional studies suggested that a healthy diet reduced the risk for depression [2, 3]. Although there are fewer prospective studies, some prospective studies also reported a healthy diet reduced the risk of depression [4, 5]. However, some other studies found no association between diet and depression [6].

Additional studies reported an association between oxidative stress and depression [7–9]. However, it remains unknown whether oxidative stress is a consequence of an unhealthy diet, of depression itself, or of both of these. Thus, recent studies proposed that depression can be treated by antioxidants [10]. In particular, a randomized placebo-controlled trial found that N-acetyl-cysteine significantly reduced depression [10].

It should be noted that a healthy diet provides significant antioxidants that can reduce oxidative stress. Moreover, a healthy diet provides a source of tryptophan and inositol, which could be important for the synthesis of neurotransmitters [11], and function as secondary messengers in numerous signal transduction pathways [12]. Legumes are important sources of tryptophan, magnesium, and inositol hexaphosphate, also known as phytate (which is partly transformed into inositol in the intestine) [13]. On the other hand, some dietary components, such as refined sugars, can induce oxidative stress and appear to increase the risk of depressive behaviors [14–16].

There is also evidence that high adherence to the Mediterranean Diet (which has high amounts of fruits, vegetables, whole-grains, legumes, and nuts) is associated with a lower risk of depressive symptoms, particularly in men [17].

In the present retrospective observational study (from 2013 to 2017), we examined the relationship of consumption of different food groups (mainly containing antioxidants, tryptophan, inositol, refined sugars) with depression. Our hypothesis was that depression could be associated to the consumption of certain food groups.

## Methods

This was an observational retrospective study of individuals admitted to the Psychology and Neurology Center (CLONUS, Mallorca, Spain) from 2013 to 2017. All individuals responded to a depression questionnaire and a simple dietary questionnaire. We examined the records of 56 individuals with no depressive levels (33 women and 23 men) and 76 individuals with depressive symptoms (53 women and 23 men). The mean (±SD) age was 41.1 (±12.9) years in the non-depressed group and 38.7 (±11.0) years in the depression group. The two groups had no significant differences in sex or age.

These individuals were recruited by CLONUS among patients and volunteers that accomplished the following inclusion and exclusion criteria.

1. Patients with severe mental health disorders (e.g., schizophrenia, major depression, bipolar disorder, and obsessive-compulsive disorder) were excluded. Only patients with a diagnostic of anxiety, depressive disorders, marital conflicts, or behavioral problems were included.
2. Participants with eating disorders were excluded.
3. Participants consuming antioxidant supplements or omega-3 PUFAs were excluded.
4. Participants with severe health problems (e.g., cancer, serious cardiopathy) that need chronic pharmacologic treatment were excluded.
5. Participants with addiction to alcohol or drugs were excluded.

All participants were Caucasian and belonging to median-high social status (they were from medium-high income households).

### Dietary questionnaire

The data on diet were extracted from a non-validated broad questionnaire, which collected information on the consumption of different food groups (Additional file 1). In particular, this questionnaire collected information regarding the consumption of the following food groups: legumes, nuts, whole-grain foods, fruits and vegetables, chocolate, sweet foods and refined sugars. For each group, no consumption was considered as “no servings per week”, low consumption as “1 or 2 servings per week”, and high consumption as “3 or more servings per week”. Servings per week were defined considering the consumptions considered as adequate in the Mediterranean diet [18]. The questionnaire was always personally passed by the same trained person.

### Depression questionnaire

The validated Beck Depression Inventory (BDI) questionnaire was used to assess depression. Individuals with BDI scores below 10.00 were considered non-depressed, and those with scores of 10.00 or more as depressed [19].

### Statistical analyses

Each value is expressed as mean (±SD) or frequency (percentage). Patients were divided into a group with no depressive levels (BDI < 10) and a group with depressive symptoms (BDI ≥ 10). The groups were compared using an independent samples Student’s t-test for continuous variables, and a chi-square test for categorical variables. Estimated effect sizes were calculated using Cramer’s V as a magnitude of association between depression and high, low or no consumption of the food groups.

Binary logistic regression was used to calculate the crude odds ratio (OR) and the OR adjusted for age and sex (aOR) for the relationship of consumption of each selected item with depression (dependent variable). A 2-tailed *p*-value less than 0.05 was considered statistically significant. Statistical analyses were performed using SPSS 23.0 (SPSS Inc., Chicago, Illinois).

## Results

Table 1 shows the consumption of each of the different food groups by the individuals with no depressive levels and with depressive symptoms. There were significant differences in the consumption of legumes, fruits and vegetables, and sweets and refined sugars. More specifically, significantly greater percentages of depressed individuals consumed no legumes (46.1% vs. 23.3%, *p* < 0.05) and a significantly lower percentage of depressed individuals consumed 3 or more servings of fruits and vegetables per week (57.9% vs. 80.4%, *p* < 0.01). However, a higher percentage of depressed individuals consumed sweets and refined sugars (36.8% vs. 16.1%, *p* < 0.05). The two groups had no significant differences in the consumption of whole-grains, nuts and chocolate. As can be seen in Table 1, estimated effect sizes indicated a small-medium association (Cramer’s V < 0.3) between depression and low consumption of legumes, fruits and vegetables; and high consumption of sweets and refined sugar.

**Table 1:** Frequency (percentage) of food consumption in the groups with no depressive levels (Beck < 10) and with depressive symptoms (Beck ≥ 10) individuals

Diet category	No depression Beck < 10 (*N* = 56)		Depression Beck ≥ 10 (*N* = 76)		Effect size Cramer’s V Mean (95% CI)	p
Legumes						
No consume	13	(23.2%)	35	(46.1%)	0.24 (0.07–0.40)	0.026
Low consume	34	(60.7%)	33	(43.4%)		
High consume	9	(16.1%)	8	(10.5%)		
Nuts						
No consume	12	(21.4%)	29	(38.2%)	0.18 (−0.02–0.38)	0.115
Low consume	21	(37.5%)	24	(31.6%)		
High consume	23	(41.1%)	23	(30.3%)		
Cereals						
No consume	17	(30.4%)	28	(36.8%)	0.08 (−0.22–0.38)	0.641
Low consume	10	(17.9%)	10	(13.2%)		
High consume	29	(51.8%)	38	(50.0%)		
Fruits and vegetables						
No consume	8	(14.3%)	10	(13.2%)	0.30 (0.09–0.51)	0.003
Low consume	3	(5.4%)	22	(28.9%)		
High consume	45	(80.4%)	44	(57.9%)		
Sweets ans pastries						
No consume	25	(44.6%)	32	(42.1%)	0.26 (0.08–0.44)	0.013
Low consume	22	(39.3%)	16	(21.1%)		
High consume	9	(16.1%)	28	(36.8%)		
Chocolate						
No consume	23	(41.1%)	32	(42.1%)	0.14 (−0.24–0.51)	0.280
Low consume	18	(32.1%)	16	(21.1%)		
High consume	15	(26.8%)	28	(36.8%)		

Differences between groups were compared with chi-square test. Estimated effect sizes were calculated using Cramer’s V statistic which is expressed as mean (95% confidence interval)

**Fig. 1:**
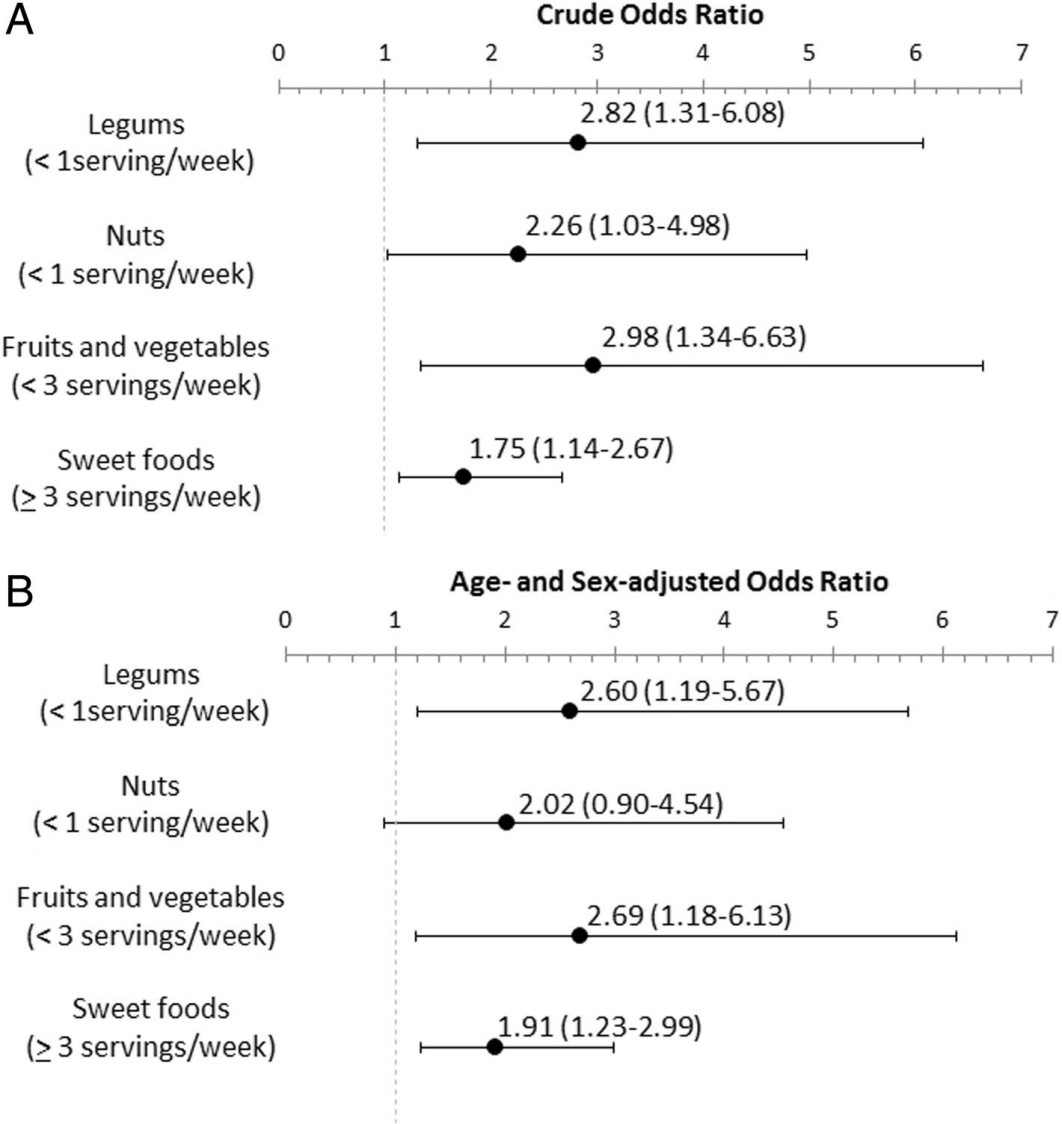
Crude (**a**) and age- and sex-adjusted (**b**) Odds Ratio of selected items of food consumption associated to depressive symptoms (Beck ≥ 10). Values are expressed as Odds Ratio (95% confidence interval)

We used binary logistic regression analysis (univariate and adjusted for age and sex) to identify the relationship of diet with depression. Univariate analysis indicated that consumption of no legumes, low consumption of fruits and vegetables (< 3 servings/weeks), and high consumption of sweets and refined sugars (≥ 3 servings weeks) were associated with depression (*p* < 0.05) (Fig. 1a). After adjusting for age and sex (Fig. 1b), consumption of no legumes (aOR = 2.60, 95% confidence interval [CI] = 1.19–5.67), low consumption of fruits and vegetables (aOR = 2.69, 95% CI = 1.18–6.13), and high consumption of sweets and refined sugars (aOR = 1.91, 95% CI = 1.23–2.99) were significantly associated with depression (Fig. 1b).

## Discussion

A finding of our study is that individuals with no depressive levels consumed more legumes. This food group is rich in tryptophan, inositol, magnesium and other important nutrients, such as fibre, folate and omega-3 fatty acids. Previous studies established a beneficial effect of consumption of tryptophan, inositol, and magnesium on the mental well-being of individuals. For example, a cross-sectional study demonstrated that higher tryptophan intake was independently associated with a lower prevalence of depression in young Japanese women [11]. Previous studies also reported lower consumption of fruits and vegetables in depressed individuals, and this is consistent with the presence of greater oxidative stress in depressed individuals [9, 20–22]. In addition, the higher consumption of sweet foods and refined sugars by depressed individuals may contribute to their increased oxidative stress. In fact, diets rich in sugars seem to induce depression [14–16]. Lipid peroxidation seems to play a role in diet-induced alterations related to behavioral disorders [14]. It is important to note that high oxidative stress can induce other pathologies, and a high level of reactive oxygen species (peroxides, superoxide, hydroxyl radical, singlet oxygen, alpha-oxygen, known as oxidative stress) is related to autoimmune responses [23, 24].

Thus, there is abundant evidence that consumption of certain foods is associated with depression and with a pathological state that leads to depression. However, further studies are necessary to determine the possible protective effect of different foods on the development of depression.

The main limitation of our study is the retrospective cross-sectional nature with a small sample, which precludes conclusions regarding the temporal nature of our findings and no solid conclusions can be established. Even though we found that the consumption of some food groups is associated to depression, we cannot confirm which one is the cause and which is the effect and also we cannot rule out a “third” explanation where there is no causal relationship between diet and depression. Another limitation is the use of a not-validated dietary food survey where the ingested amounts of each product are not specified. In addition, non-depressed individuals have been selected among patients who went to the Center of Psychology and Neurology, so they are people who, although not depressed, may suffer some kind of non-serious disease. Therefore, the group without depression is not, a group of totally healthy individuals. Also, the profession and social status have not been considered, although all the participants can be considered to belong to a medium-high social status. For all these reasons, prospective studies are needed to establish the time sequence in the relationship between them and clinically relevant findings.

## Conclusion

In spite of these limitations, we observed significant differences in the diets of individuals with no depressive levels and with depressive symptoms. In particular, individuals without depression consumed more legumes, fruits, and vegetables, but fewer sweets and pastries than those with depressive symptoms.

## Additional file

Additional file 1: Dietary Questionnaire used to obtain the information on the consumption of different food groups (Legumes, Nuts, Whole grain foods, Fruits and vegetables, Chocolate, Sweet foods and refined sugars). (DOC 45 kb)

## Abbreviations

aOR: Adjusted odds ratio
BDI: Beck Depression Inventory Questionnaire
CI: Confidence interval
DSM-5: Diagnostic and statistical manual
PUFAs: Polyunsaturated fatty acids
SPSS: Statistical package for social sciences
