# Supplementary material for: Possible relation between consumption of different food groups and depression
Source: BMC Psychol. 2019 Mar 6;7:14. doi: 10.1186/s40359-019-0292-1 (PMC6404288; doi:10.1186/s40359-019-0292-1)
Supplement: Supplementary file 1 — Dietary Questionnaire used to obtain the information on the consumption of different food groups (Legumes, Nuts, Whole grain foods, Fruits and vegetables, Chocolate, Sweet foods and refined sugars). (DOC 45 kb) [file 40359_2019_292_MOESM1_ESM.doc]

**DIETARY QUESTIONNAIRE**

Information on the consumption of different food groups

| **LEGUMES** | No servings per week |  |
| --- | --- | --- |
| (white beans, chickpeas, broad beans, | 1-2 servings per week |  |
| green peas) | 3 or more servings per week |  |
|  |  |  |
| **NUTS** | No servings per week |  |
| (almonds, cashew nuts, hazelnuts, | 1-2 servings per week |  |
| walnuts, peanuts) | 3 or more servings per week |  |
|  |  |  |
|  |  |  |
| **WHOLE GRAIN FOODS** | No serving per week |  |
| (whole wheat bread, oatmeal, whole | 1-2 servings per week |  |
| grain cereal, whole wheat pasta) | 3 or more servings per week |  |
|  |  |  |
|  |  |  |
| **FRUITS AND VEGETABLES** | No serving per week |  |
|  | 1-2 servings per week |  |
|  | 3 or more servings per week |  |
|  |  |  |
|  |  |  |
| **CHOCOLATE** | No serving per week |  |
|  | 1-2 servings per week |  |
|  | 3 or more servings per week |  |
|  |  |  |
|  |  |  |
| **SWEET FOODS AND REFINED** | No servings per week |  |
| **SUGARS** | 1-2 servings per week |  |
| (cake, cookies, doughnuts, ice creams | 3 or more servings per week |  |
| Industrial pastry, honey) |  |  |
